# Modulation of Cytokeratin Expression in The Hamster Thymus: Evidence For a Plasticity of The Thymic Epithelium

**DOI:** 10.1155/1993/94342

**Published:** 1993

**Authors:** Lucia Renata Meireles De Souza, Wilson Savino

**Affiliations:** Department of Immunology Institute Oswaldo Cruz-FIOCRUZ Rio de Janeiro Brazil

**Keywords:** Thymic epithelium, hamster, cytokeratins, *in vitro*, thymus ontogeny, hydrocortisone

## Abstract

Cytokeratin (CK) expression was investigated, by means of immunocytochemistry, in the hamster thymic epithelium during ontogeny, as well as in primary cultures and upon glucocorticoid hormone treatment *in vivo*. As compared to the distribution pattern of distinct monoclonal antibody-defined cytokeratins in the normal adult thymus, CK modulation was evidenced in the three situations studied. During thymus ontogeny, both cytokeratins of simple lining epithelia, as CK8 and CK18, as well as the CK1/CK10 pair (typical marker of terminal stage of keratinization), were expressed since early stages of thymus development. They were located in the central region of thymic lobules preceding the cortical-medullary distinctions. This differed from what had been previously shown for mouse thymus ontogeny, revealing that the interspecific diversity in the distribution pattern of thymic cytokeratins occurred early in fetal life. A modulation of CK expression was also detected when hamster thymic epithelial cells (TEC) were led to grow in culture, with a down-regulation of CK19 contrasting with an enhancement of CK18 expression. This diverged from the maintenance of the *in situ* pattern when human TEC were cultured. Last, *in vivo* hydrocortisone treatment, known to increase the numbers of KL1^+^ cells in the mouse thymus medulla, promoted a cortical expression of the CK1/CK10 pair in the hamster thymus. Taken together, our findings demonstrate a continuous plasticity of the thymic epithelium, at least regarding cytokeratin expression, and enlarge the concept of interspecific diversity of intrathymic CK distribution in conditions as morphogenesis, *in vitro* system, and responsiveness to glucocorticoid hormone treatment.


*Developmental Immunology*, 1993, Vol. 3, pp. 137–146
Reprints available directly from the publisher
Photocopying permitted by license only

© 1993 Harwood Academic Publishers GmbH
Printed in the United States of America

### Modulation of Cytokeratin Expression in the Hamster Thymus: Evidence for a Plasticity of the Thymic Epithelium

LUCIA RENATA MEIRELES DE SOUZA and WILSON SAVINO*

*Department of Immunology, Institute Oswaldo Cruz–FIOCRUZ, Rio de Janeiro, Brazil*

Cytokeratin (CK) expression was investigated, by means of immunocytochemistry, in the hamster thymic epithelium during ontogeny, as well as in primary cultures and upon glucocorticoid hormone treatment *in vivo*. As compared to the distribution pattern of distinct monoclonal antibody-defined cytokeratins in the normal adult thymus, CK modulation was evidenced in the three situations studied. During thymus ontogeny, both cytokeratins of simple lining epithelia, as CK8 and CK18, as well as the CK1/CK10 pair (typical marker of terminal stage of keratinization), were expressed since early stages of thymus development. They were located in the central region of thymic lobules preceding the cortical-medullary distinctions. This differed from what had been previously shown for mouse thymus ontogeny, revealing that the interspecific diversity in the distribution pattern of thymic cytokeratins occurred early in fetal life. A modulation of CK expression was also detected when hamster thymic epithelial cells (TEC) were led to grow in culture, with a down-regulation of CK19 contrasting with an enhancement of CK18 expression. This diverged from the maintenance of the *in situ* pattern when human TEC were cultured. Last, *in vivo* hydrocortisone treatment, known to increase the numbers of $KL1^+$ cells in the mouse thymus medulla, promoted a cortical expression of the CK1/CK10 pair in the hamster thymus. Taken together, our findings demonstrate a continuous plasticity of the thymic epithelium, at least regarding cytokeratin expression, and enlarge the concept of interspecific diversity of intrathymic CK distribution in conditions as morphogenesis, *in vitro* system, and responsiveness to glucocorticoid hormone treatment.

KEYWORDS: Thymic epithelium, hamster, cytokeratins, *in vitro*, thymus ontogeny, hydrocortisone.

### INTRODUCTION

Studies on thymocyte differentiation are often carried out on fetal specimens or *in vitro*, providing important information for the role of the thymic microenvironment in this process. Nonetheless, to evaluate the function of each microenvironmental component, it is essential that the markers known to recognize it in the adult thymus *in situ* could also be used in other experimental systems. A large number of monoclonal antibodies (MAbs) was postulated to be markers of thymic epithelial cell (TEC) subsets (Haynes, 1984; van Vliet et al., 1984; de Maagd et al., 1985; Kaneshima et al., 1987; Takacs et al., 1987; Izon and Boyd, 1990). Yet, no specific functional subset was isolated so far, despite the several TEC lines produced (Itoh, 1979; Nieburgs et al., 1985; Potworowski et al., 1986; Mizutani et al., 1987; Naquet et al., 1989).

Interestingly, these markers reveal a close antigenic similarity between the thymic epithelium and epidermis (Haynes, 1984; Lobach et al., 1985; Schmitt et al., 1987), suggesting that TEC heterogeneity could also be a reflex of their type of differentiation, as demonstrated for the epidermis. In this regard, van de Wijngaert et al. (1984) proposed a TEC differentiation process occurring essentially in the cortical region, yet participating in Hassall's corpuscle formation. This hypothesis contrasted to that previously raised by Mandel (1968) postulating that Hassall's corpuscle rose from some medullary TEC. In this respect, many

*Corresponding author.

anti-TEC MAb reacting to Hassall's corpuscles and few surrounding medullary TEC recognize the *stratum corneum* (Haynes, 1984; Lobach et al., 1985); other MAb, pan-specific to the thymic epithelium (including Hassall's corpuscles), label all epidermal sheets, whereas MAb recognizing the whole thymic epithelium network, but Hassall's corpuscles restrictly stain epidermal basal layers (Schmitt et al., 1987). It was then postulated that Hassall's corpuscles might correspond to a final degree of TEC differentiation, as the *stratum corneum* keratinocytes. In this context, anticytokeratin (anti-CK) MAb, previously defined as epithelial differentiation markers might be regarded as relevant tools in the study of TEC subsets, as evidenced in rodents and humans (Nicolas et al., 1985, 1986, 1989; Laster and Haynes, 1986; Colic et al., 1988a, 1988b; Savino and Dardenne, 1988a, 1988b; Farr and Brady, 1989).

The set of cytokeratins expressed by an epithelium is typical of this tissue and related to its differentiation (Moll et al., 1982a; Quinlan et al., 1985; Sun et al., 1985). Nonetheless, concerning the thymic epithelium, we evidenced the expression of cytokeratins both of simple and stratified epithelia, as well as an interspecific diversity in the distribution of these intermediate filament proteins. These results suggested that the thymic epithelium is complex and unique, and that the general classification of epithelial tissues based on their CK expression patterns cannot be applied to the thymus (Meireles de Souza et al., 1993).

In the present work, we used the hamster as a model to study if in a given species, the *in situ* pattern could be reproduced in other experimental situations, which seems to be essential for a further evaluation on the precise role of distinct thymic microenvironmental components.

We addressed the question concerning TEC differentiation and CK expression, using the hamster model during thymic ontogeny, primary TEC cultures, and following hydrocortisone treatment, the latter known to augment KL1-defined CK expression in the mouse thymus (Savino et al., 1988). It is important to note that the hamster thymus presents a late cortical-medullary distinction (1 week after birth), although the appearance of Hassall's corpuscles precedes this distinction (Adner et al., 1965), making this species particularly interesting.

We show herein that CK expression in the hamster thymus can be modulated in the three

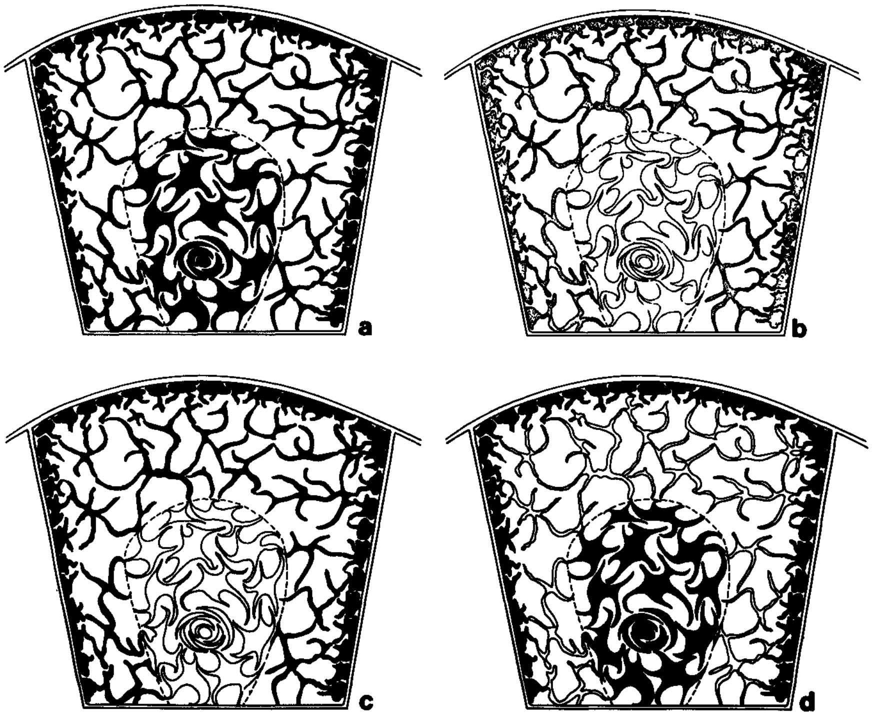

FIGURE 1. Coexpression of cytokeratins typical of simple and stratified epithelia in the adult hamster thymus. Panel (a) shows the whole thymic epithelial network expressing CK19. Panels (b) and (c), respectively, reveal CK8 and CK18 labeling. Although both anti-CK MAb stain the same cortical zone, it is noteworthy that TEC are rather weakly stained by the anti-CK9 MAb. Panel (d) shows KL1 staining in both subcapsullary and medullary zones (×160).

situations analyzed, thus evidencing a continuous plasticity of thymic epithelial cells. These data suggest that TEC heterogeneity depends on the developmental stage, species of origin, hormonal status, and *in situ/in vitro* systems. Lastly, our findings indicate that the thymic epithelium may not be divided in definitive subsets, and that phenotypic markers of TEC heterogeneity should be considered as markers to trace TEC plasticity.

### RESULTS

### Cytokeratin Expression During Hamster Thymus Ontogeny

As reported in Meireles de Souza et al. (1993), the adult hamster thymus presented the coexpression of CK typical of simple and stratified epithelia (Fig. 1). Interestingly, this was already observed since day 13 of fetal life (Fig. 2). In fact, at early stages of thymus ontogeny, $CK8^+$, $CK18^+$, and $KL1^+$ cells were predominantly located in the central region of the thymic lobes. Cortical and medullary TEC were distinguished by anti-CK MAb solely at the time of cortical-medullary distinction. It is noteworthy that the predominance of CK18 expression observed in the adult hamster, as compared to CK8 distribution, was stressed during ontogeny.

Regarding the expression of CK19, we noticed that the whole thymic epithelium was already stained by the anti-CK19 MAb on the 13th day of fetal age (Fig. 2b). Actually, the anti-CK19 MAb was the only reagent that reacted similarly during fetal or adult age. In contrast, CK13, which is expressed by medullary/subcapsullary TEC in the adult thymus, was not detected during ontogeny, even in animals of age 1 week of postnatal life.

On the 15th fetal day, thymus lobulation was initiated, and we could detect subseptal TEC intensely labeled by anti-CK19 MAb. Lastly, the appearance of Hassall's corpuscles at birth was clearly defined by KL1 staining (Fig. 3).

The acquisition and compartmentalization of cytokeratin expression during the hamster thymus ontogeny are summarized in Fig. 4.

### Cytokeratin Expression in Hamster Thymus Primary Cultures

Because an ontogenetic-dependent plasticity of CK expression in hamster TEC appeared to occur, we asked the question whether such modulation could also take place *in vitro*. The

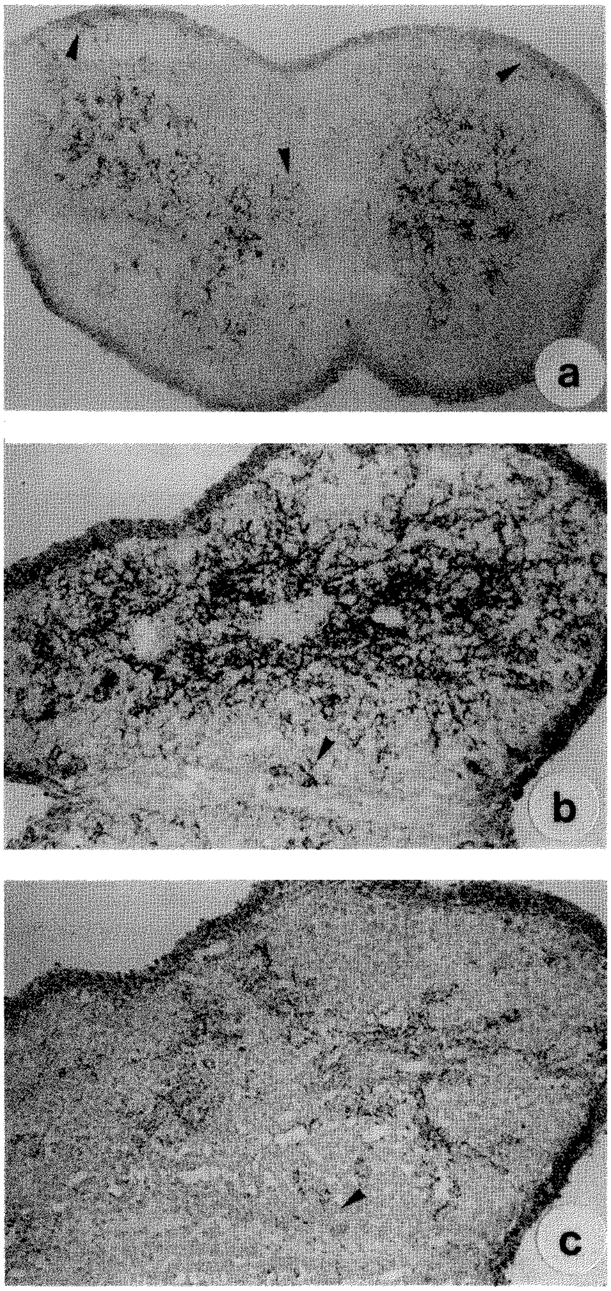

FIGURE 2. Coexpression of cytokeratins typical of simple and stratified epithelia in the 13-day fetal thymus of hamster. Panel (a) depicts $CK8^+$ TEC being predominantly found in the central region of thymic lobules (×160). In panel (b), the whole epithelial network was labeled by the anti-CK19 MAb (×250), whereas in panel (c) (showing the same thymic lobule seen in (b)), less cells in the central region are stained with KL1 (×250).

rationale for that was based on our previous findings showing that human primary TEC cultures revealed a CK pattern similar to that found *in situ* (Savino and Dardenne, 1988b). Nonetheless, the hamster thymic epithelium presented a CK pattern largely different from that found *in vivo* (Fig. 5). Although anti-CK19 was a pan-TEC marker *in situ*, a rather low percentage of cells (20–40%) in the hamster thymus primary cultures was labeled. Actually, the cytokeratin predominantly

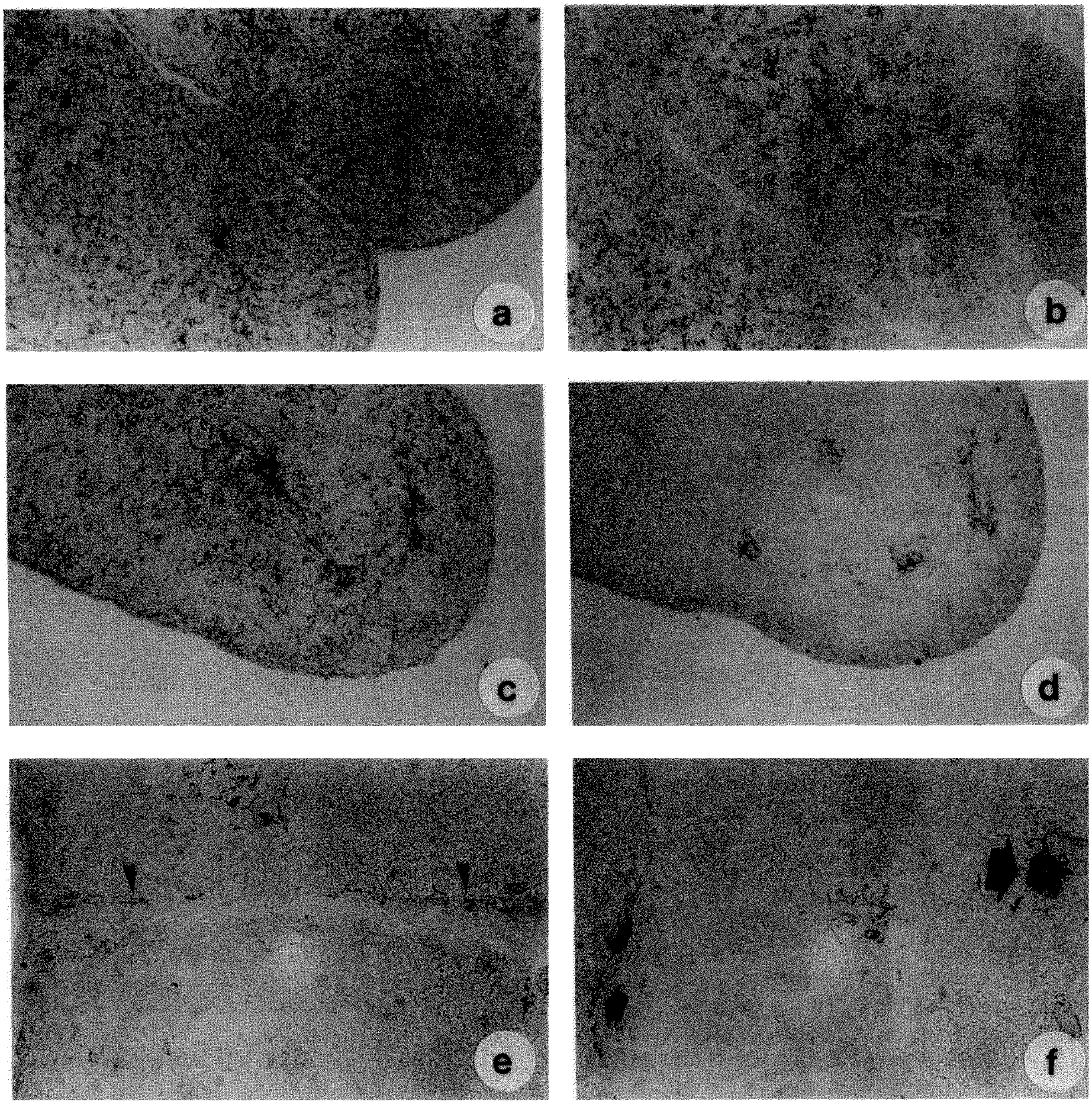

FIGURE 3. Cytokeratin expression pattern in the neonatal hamster thymus. In panel (a), the whole TEC network is seen labeled by the anti-CK19 MAb (×100). Panel (b) shows the same thymic region stained with the anti-CK18 MAb, revealing that CK18⁺ TEC are predominantly found in the central region (×100). CK19-expressing TEC network is seen in panel (c) (×160), whereas in the same region, KL1⁺ TEC are restricted to the central region (panel (d); ×160). Additionally, panel (e) reveals KL1⁺ subseptal TEC (arrowheads; ×400), and panel (f) shows KL1⁺ Hassall's corpuscles (large arrow) and subseptal TEC (short arrow; ×400).

expressed *in vitro* was CK18 (>80%), with CK8 expression being consistently lower, in parallel to what was observed during ontogeny. Regarding CK1/CK10 expression, immunocytochemically defined by KL1 MAb, only a minor percentage of TEC (1–5%) was KL1-reactive.

Interestingly, the previously described *in vitro* pattern was maintained in cultures of 1, 2, or 3 weeks. Moreover, it was not altered by hydrocortisone addition at the doses of $10^{-3}$, $10^{-5}$, or $10^{-7}$ M, previously known to increase the numbers of $KL1^+$ cells in a mouse TEC line (Savino et al., 1988). As summarized in Table 1, the *in vitro* pattern of CK expression by hamster TEC differs from that described for human TEC cultures (Savino and Dardenne, 1988b).

### *In Vivo* Modulation of CK Expression by Hydrocortisone

In a third group of experiments, we probed the responsiveness of hamster thymic microenvironment following hydrocortisone treatment *in vivo*. This strategy promoted an increase in the numbers of $KL1^+$ cells in mice (Savino et al., 1988), as well as in the expression of extracellular matrix proteins (Lannes-Vieira et al., 1991).

Regarding KL1 reactivity, besides the normal subcapsullary-medullary staining, cortical TEC were also recognized by this MAb in hydrocorti-

TABLE 1
Comparative *In Vitro* Cytokeratin Expression Patterns[a] in Hamster and Human Cultures of Thymic Epithelial Cells

| Anti-CK antibodies | Hamster | Human[b] |
|---|---|---|
| Anti-CK8 | + | NT[c] |
| Anti-CK18 | +++ | + |
| Anti-CK19 | ++ | +++ |
| KL1 | + | +++ |

[a]Ascertained by percentage of labeled cells with each anti-CK MAb: +=1–5%; ++=20–40%; and +++>80%.
[b]Data from Savino and Dardenne (1988b).
[c]NT: not tested.

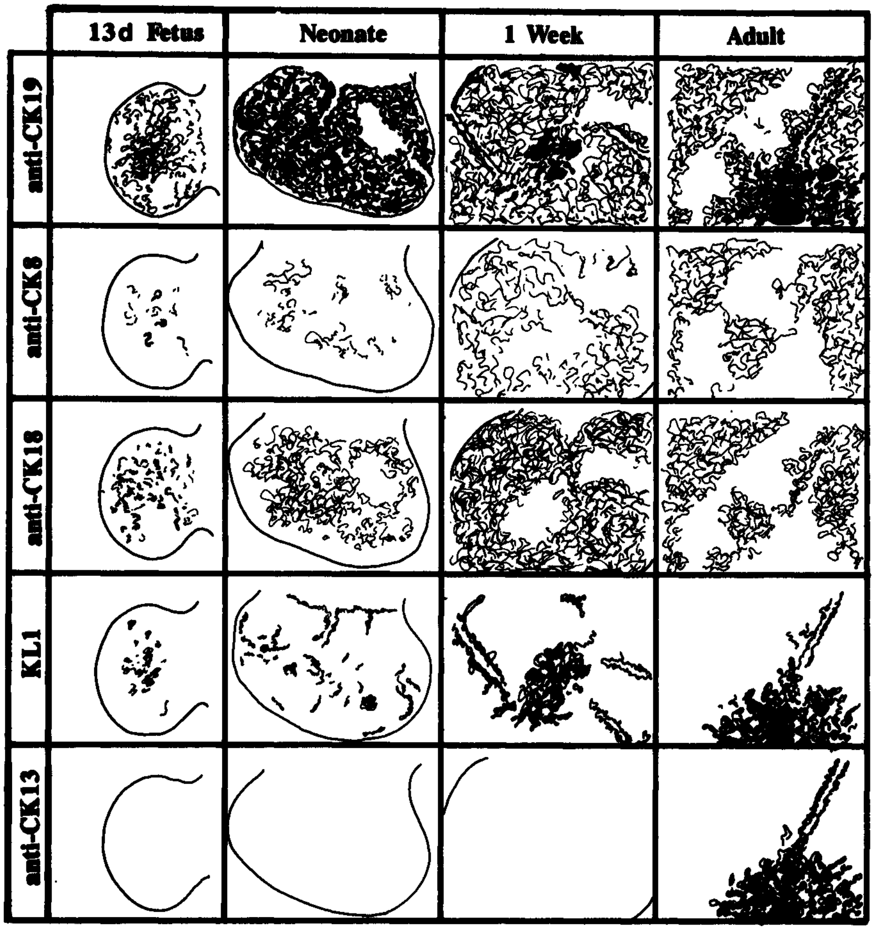

FIGURE 4. The acquisition and compartmentalization of distinct cytokeratins along with hamster thymus ontogeny.

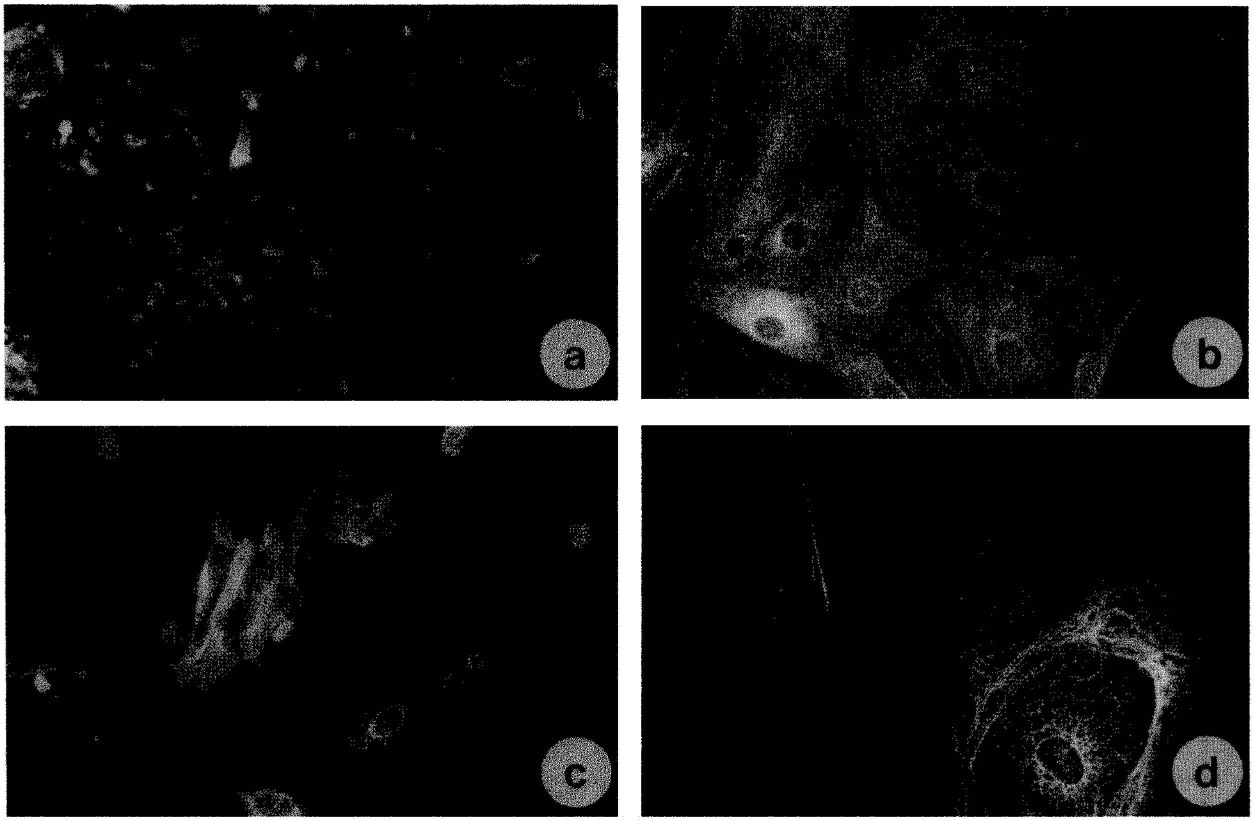

FIGURE 5. Immunofluorescence of hamster thymic primary cultures revealing TEC morphological heterogeneity *in vitro* as detected in panel (a) by the pan-specific antikeratin polyclonal antibody HTK (×125). Panel (b) depicts a cluster of polygonal TEC, also stained with HTK (×320), whereas in panel (c), CK19$^+$ TEC are seen (×320), and the intracytoplasmatic CK18$^-$ containing intermediate filament network is shown in panel (d) (×320).

sone-treated animals (Fig. 6a). Additionally, CK8 and CK18 expression (normally restricted to the cortex in young adult hamster thymus) was also detected in peripheral TEC of Hassall's corpuscles and some scattered medullary TEC (Fig. 6b).

Interestingly, concerning the other microenvironmental compartment, namely, the extracellular matrix component (whose distribution was demonstrated to be rather conserved in mammals; Meireles de Souza et al., 1993), the expression of fibronectin, laminin, and type-IV collagen was extended to the cortical region of hamster thymus (Fig. 7), similarly to that described in hydrocortisone-treated mice (Lannes-Vieira et al., 1991).

### DISCUSSION

In the present work, we bring consistent data showing the modulation of cytokeratin expression by hamster thymic epithelial cells, following three distinct biological situations. As discussed in detail in what follows, the findings presented herein indicate a continuous plasticity of the thymic epithelium, at least in terms of intermediate filament proteins.

The first aspect to be considered regards CK expression during hamster thymus ontogeny. In relatively early fetal stages at day 13, it was noteworthy that both CK8/CK18 and CK1/CK10 pairs, respectively, markers of simple and stratified highly keratinized lining epithelia, were expressed in the central region of the thymic lobes. This simultaneous CK expression largely preceded the cortical-medullary distinction, which occurs 1 week after birth (Adner et al., 1965).

The results on KL1 staining contrasted with the data previously reported for the mouse thymus ontogeny (Savino and Dardenne, 1988a) in which KL1$^+$ cells appeared later. In this regard, the concept for an interspecific diversity in the thymus

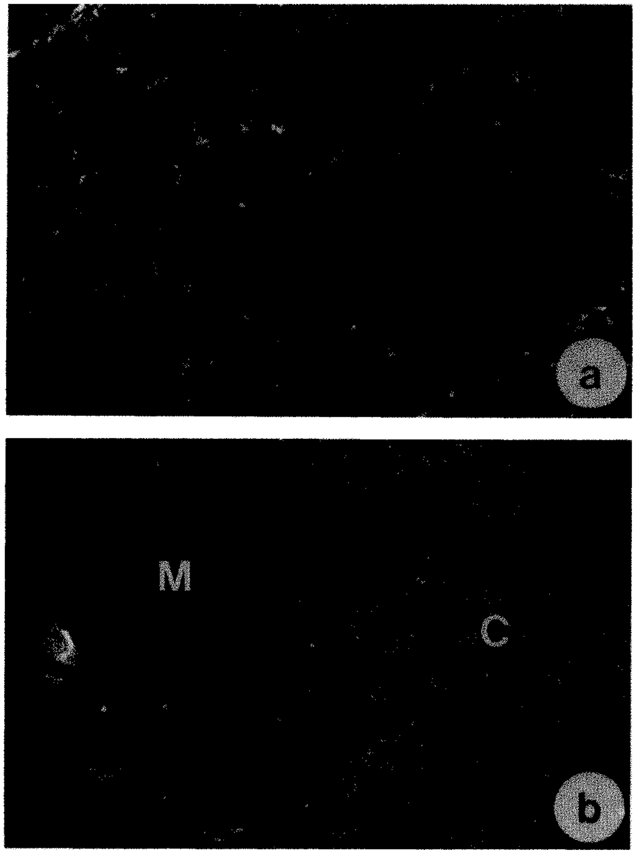

FIGURE 6. Immunofluorescence of hamster thymus sections 24 hr after *in vivo* hydrocortisone treatment labeled with KL1 and anti-CK18 MAb, respectively, in panels (a) and (b). $KL1^+$ TEC can be seen in the cortical region, whereas CK18 staining is found in a Hassall's corpuscle (arrow) and cortical TEC (× 320): C: cortex.

CK distribution should also comprise ontogeny. Moreover, because in the adult thymus, the localization of these CK pairs is different, it is likely to occur as down- and up-regulation of CK expression along with the definition of the adult pattern. This is further supported because CK13 is acquired much later during hamster development, whereas some weak CK13 reactivity was detected from day 17 of fetal life to 1 week after birth in the rat thymus, and then was absent in the adult organ (Colic et al., 1990).

The second point deserving discussion concerns the modulation of CK expression in hamster TC cultures. This was clearly evidenced by the analysis of CK19 labeling. Although the anti-CK19 MAb stained the whole TEC network *in situ*, less than half of cultured cells were reactive. In contrast, there was a predominance of $CK18^+$ TEC, with TEC expressing CK18 but not CK19. This may not be the result of cortical TEC selection *in vitro* (at least as they are phenotyped *in situ*), because the hamster thymic cortex is $CK18^+$, $CK8^+$, and $CK19^+$. Nevertheless, such decrease in CK19 expression may be due rather to culture conditions used herein, which may be suitable for human but not for hamster primary cultures in terms of keeping CK19 expression.

In any case, this *in vitro* pattern did not significantly change even in 3-week-old cultures. In this respect, $KL1^+$ cells, expected to represent the terminal stage of TEC differentiation, actually remained as a minor population, thus differing from the *in vitro* differentiation process reported for a rat TEC line (Itoh et al., 1982; Lobach and Haynes, 1987). Furthermore, the hydrocortisone-induced increase in $KL1^+$ cells that occurs in mouse TEC line (Savino et al., 1988) was not detected in hamster cultures. Thus, acquisition of high-molecular-weight CKs, typical of epithelial differentiation, did not occur in the hamster model *in vitro*. In addition, the results obtained with cultured hamster TEC, when compared to those reported for human TEC cultures, again point out an interspecific diversity of CK expression even when *in vitro* conditions are considered.

Besides the modulation of CK expression by hamster TEC during thymus ontogeny and *in vitro* conditions, the *in vivo* treatment with hydrocortisone significantly changed the distribution pattern of distinct cytokeratins. $CK18^+$ cells (normally restricted to the cortex) also appeared in the medulla (adjacent to Hassall's corpuscles), whereas KL1-defined CK expression was enhanced, being evidenced throughout the cortex. A modulation of $KL1^+$ cells upon a single dose of hydrocortisone was previously observed in the mouse thymus (Savino et al., 1988). Nonetheless, in this species, the medullary restriction of these cells was maintained. Thus, although a plasticity in the expression of a KL1-defined CK pair occurred in both species following this experimental condition, an interspecific diversity was again evidenced. In contrast, the responsiveness concerning the expression of extracellular matrix proteins (whose normal distribution is conserved in mammals) was similar to that observed in mice (Lannes-Vieira et al., 1991).

In conclusion, the data presented and dis-

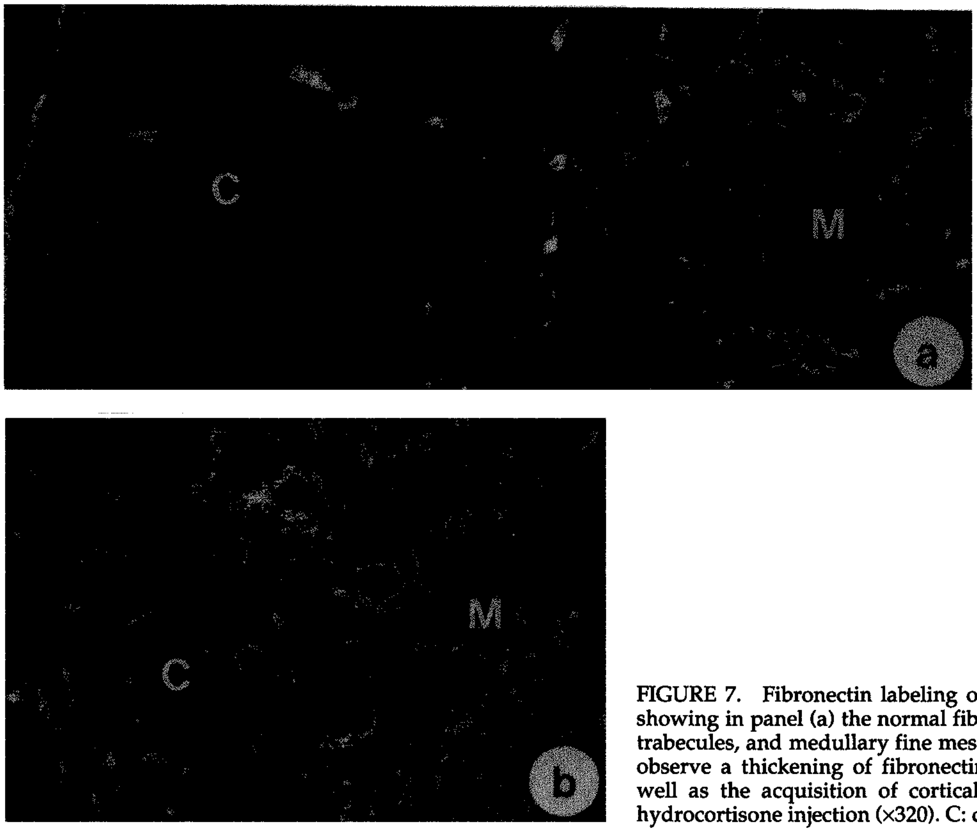

FIGURE 7. Fibronectin labeling of hamster thymus sections, showing in panel (a) the normal fibronectin pattern in capsule, trabecules, and medullary fine meshwork. In panel (b), we can observe a thickening of fibronectin medullary meshwork, as well as the acquisition of cortical fibers, 24 hr after *in vivo* hydrocortisone injection (×320). C: cortex; M: medulla.

cussed herein clearly demonstrate the continuous plasticity of the thymic epithelium, at least regarding expression of intermediate filament proteins. In this context, anti-CK monospecific antibodies can be ascribed as useful tools to define such plasticity, even when rather subtle modulations are involved, as we previously demonstrated in human thymomas (Savino and Dardenne, 1988b), murine Chaga's disease (Savino et al., 1989), and in the nonobese diabetic mouse (Savino et al., 1991). In this respect, our findings suggest that the thymic epithelium may not be divided in definitive subsets, and that phenotypic markers of TEC heterogeneity should be rather considered as markers to trace TEC plasticity.

### MATERIALS AND METHODS

### Thymuses

Outbred hamsters *(Mesocricetus auratus)* were obtained from the animal house of the Oswaldo Cruz Foundation (Rio de Janeiro), and the inbred CB and LHC strains were provided by the animal facilities of the Department of Microbiology (Federal University of Rio de Janeiro).

### Antibodies

A variety of anti-CK and anti-ECM antibodies was used in this study, and the characteristics are detailed in Meireles de Souza et al. (1993). Briefly, we applied pan-CK markers, respectively, HTK and KL4, as well as monospecific anti-CK reagents recognizing CK8, CK18, CK19 (found in simple epithelia), and CK13 and the CK1/CK10 pair (typical of stratified epithelia). With regard to ECM reagents, we used rabbit polyclonal antisera specifically recognizing laminin, fibronectin, or type-IV collagen (Institut Pasteur, Lyon). The distribution of these proteins in the normal thymus have been described previously for humans (Berrih et al., 1985), mice (Lannes-Vieira et al., 1991), and a variety of mammals (Meireles de Souza et al., 1993). Secondary antibodies used herein were also described in Meireles de Souza et al. (1993).

### Immunohistochemistry

Acetone-fixed, 4-$\mu$m thymus sections or methanol-fixed cell cultures were subjected to indirect immunofluorescence or immunoperoxidase assays. Description of these techniques can be found in Meireles de Souza et al. (1993).

### Primary Cultures of Hamster Thymus

Primary cultures of hamster thymic nonlymphoid cells were carried out according to the technique originally described by Papiernik et al. (1975). Briefly, thymuses were minced by scissors, and 1-mm$^3$ fragments were incubated in MEM DVal supplemented with 10% fetal calf serum, 10-mM HEPES, 2-mM glutamine, 100-U/ml penicillin, and 100-$\mu$g/ml streptomycin at 5% $CO_2$ atmosphere, using 25-ml tissue culture flasks (Nunc Co., Roskilde, Denmark).

We chose the MEM DVal medium because it favors the epithelial cell proliferation in comparison to fibroblast growth (Gilbert and Migeon, 1975). Culture medium was changed twice a week, and cells were analyzed on days 7, 14, and 21 of culture.

### Hydrocortisone Treatment

Primary cultures of hamster TEC were treated with sodic hemisuccinate of hydrocortisone (Sigma Co., St. Louis, MO), with doses ranging from $10^{-3}$ to $10^{-7}$ M. Cultures were fixed in absolute methanol on days 7 and 14, and evaluated for CK expression by immunofluorescence assay. When *in vivo* experiments were carried out, young adults were injected i.p. with a single dose of hydrocortisone sodic hemisuccinate (10 mg/20 g of body weight), being sacrificed 24 hr later. Thymuses were excised, snap frozen into liquid nitrogen, and processed for immunohistochemistry.

### ACKNOWLEDGMENTS

This work was partially supported by the Brazilian Research Council (CNPq) and the Commission of the European Communities (CEC).

*(Received August 25, 1992)*

*(Accepted September 19, 1992)*

### REFERENCES

Adner M.M., Sherman J.D., and Dameshek W. (1965). The normal development of the lymphoid mass in the golden hamster and its relationship to the effects of thymectomy. Blood **25:** 511–521.

Berrih S., Savino W., and Cohen S. (1985). Extracellular matrix of the human thymus: Immunofluorescence studies on frozen sections and cultured epithelial cells. J. Histochem. Cytochem. **33:** 655–664.

Colic M., Matanovic D., Hegedis L., and Dujic A. (1988a). Immunohistochemical characterization of rat thymic nonlymphoid cells. I. Epithelial and mesenchymal components defined by monoclonal antibodies. Immunology **65:** 277–284.

Colic M., Matanovic D., Hegedis L., and Dujic A. (1988b). Heterogeneity of rat thymic epithelium defined by monoclonal anti-keratin antibodies. Thymus **12:** 123–130.

Colic M., Dragojevic-Simic V., Gasic S., and Dujic A. (1990). Interspecies differences in expression of cytokeratin polypeptides within thymic epithelium: A comparative immunohistochemical study. Develop. Comp. Immunol. **14:** 347–354.

de Maagd R.A., Mackenzie W.A., Schuurman H.-J., Ritter M.A., Price K.M., Broekhuizen R., and Kater L. (1985). The human thymus microenvironment: Heterogeneity detected by monoclonal anti-epithelial antibodies. Immunology **54:** 745–754.

Farr A.G., and Brady S.C. (1989). Patterns of keratin expression in the murine thymus. Anat. Rec. **224:** 374–378.

Gilbert S.F., and Migeon B.R. (1975). D-Valine as a selective agent for normal human and rodent epithelial cells in culture. Cell **5:** 11–17.

Haynes B.F. (1984). The human thymic microenvironment. Adv. Immunol. **36:** 87–142.

Itoh T. (1979). Establishment of an epithelial cell line from rat thymus. Am. J. Anat. **156:** 99–107.

Itoh T., Kasahara S., Aizu S., Kato K., Takeuchi M., and Mori T. (1982). Formation of Hassall's Corpuscles *in vitro* by the thymic epithelial cell line IT26R21 of the rat. Cell Tissue Res. **226:** 469–475.

Izon D.J., and Boyd R.L. (1990). The cytoarchitecture of the human thymus detected by monoclonal antibodies. Human Immunol. **27:** 16–32.

Kaneshima H., Ito M., Asai J., Taguchi O., and Hiai H. (1987). Thymic epithelial reticular cell subpopulations in mice defined by monoclonal antibodies. Lab. Invest. **56:** 372–380.

Lannes-Vieira J., Dardenne M., and Savino W. (1991). Extracellular matrix components of the mouse thymic microenvironment. Ontogenic studies and modulation by glucocorticoid hormones. J. Histochem. Cytochem. **39:** 1539–1546.

Laster A.H., and Haynes B.F. (1986). Characterization of a monoclonal antibody, RTE-21, that binds to keratohyalin granule-associated proteins in epithelial cells of human skin and thymus. Clin. Immunol. Immunopathol. **41:** 130–144.

Lobach D.F., and Haynes, B.F. (1987). Ontogeny of the human thymus during fetal development. J. Clin. Immunol. **7:** 81–97.

Lobach D.F., Scearce R.M., and Haynes B.F. (1985). The human thymic microenvironment. Phenotypic characterization of Hassall's bodies with the use of monoclonal antibodies. J. Immunol. **134:** 250–257.

Mandel T. (1968). The development and structure of Hassall's corpuscles in the guinea pig. A light and electron microscopic study. Z. Zellforsch. **89:** 180–192.

Meireles de Souza L.R., Trajano V., and Savino W. (1993). Is there an interspecific diversity of the thymic microenvironment. Develop. Immunol.

Mizutani S., Watt S.M., Robertson D., Hussein S., Healy L.E., Furley A.J.W., and Greaves M.F. (1987). Cloning of human thymic subcapsular cortex epithelial cells with T-lymphocyte binding sites and hemopoietic growth factor activity. Proc. Natl. Acad. Sci. USA **84:** 4999–5003.

Moll R., Franke W.W., and Schiller D.L. (1982). The catalog of human cytokeratins: Patterns of expression in normal epithelia, tumors and cultured cells. Cell **31:** 11–24.

Naquet P., Lepesant H., Luxembourg A., Brekelmans P., Devaux C., and Pierres M. (1989). Establishment and characterization of mouse thymic epithelial cell lines. Thymus **13:** 217–227.

Nicolas J.-F., Reano A., Kaiserlian D., and Thivolet J. (1986). Epithelial cell heterogeneity in the guinea pig thymus: Immunohistochemical characterization of four thymic epithelial subsets defined by monoclonal anti-keratin antibodies. Eur. J. Immunol. **16:** 457–464.

Nicolas J.-F., Reano A., Kaiserlian D., and Thivolet J. (1989). Epithelial cell heterogeneity in mammalian thymus: Monoclonal antibody to high molecular weight keratins exclusively binds to Hassall's corpuscles. Histochem. J. **21:** 357.

Nicolas J.-F., Savino W., Reano A., Viac J., Brochier J., and Dardenne M. (1985). Heterogeneity of thymic epithelial cell (TEC) keratins. Immunohistochemical and biochemical evidence for a subset of highly differentiated TEC in the mouse. J. Histochem. Cytochem. **33:** 687–694.

Nieburgs A.C., Picciano P.T., Korn J.H., McCalister T., Allred C., and Cohen S. (1985). *In vitro* growth and maintenance of two morphologically distinct populations of thymic epithelial cells. Cell. Immunol. **90:** 439–450.

Papiernik M., Nabarra B., and Bach J.F. (1975). *In vitro* culture of functional human thymic epithelium. Clin. Exp. Immunol. **19:** 281–287.

Potworowski E.F., Turcotte F., Beauchemin C., Hugo P., and Zelechowska M.G. (1986). Establishment and characterization of a thymic medullary epithelial cell clones. In Vitro Cell. & Develop. Biol. **22:** 557–560.

Quinlan R.A., Schiller D.L., Hatzfeld M., Achtstatter T., Moll R., Jorcano J.L., Magin T.M., and Franke W.W. (1985). Patterns of expression and organization of cytokeratin intermediate filaments. Ann. N.Y. Acad. Sci. **455:** 282–305.

Savino W., Boitard C., Bach J.F., and Dardenne M. (1991). Studies on the thymus in nonobese diabetic (NOD) mice. I. Changes in the microenvironment compartments. Lab. Invest. **64:** 405–417.

Savino W., Cirne-Lima E.O., Soares J.F.T., Leite de Moraes M.C., Ono I.P.C., and Dardenne M. (1988). Hydrocortisone increases the numbers of KL1 cells, a discrete thymic epithelial cell subset characterized by high molecular weight cytokeratin expression. Endocrinology **123:** 2557–2564.

Savino W., and Dardenne M. (1988a). Developmental studies on the expression of monoclonal antibody defined cytokeratins by thymic epithelial cells from normal and autoimmune mice. J. Histochem. Cytochem. **36:** 1123–1128.

Savino W., and Dardenne M. (1988b). Immunohistochemical studies on a human thymic epithelial cell subset defined by the anticytokeratin 18 monoclonal antibody. Cell Tissue Res. **254:** 225–231.

Savino W., Leite de Moraes M.C., Hontebeyrie-Joskowicz M., and Dardenne M. (1989). Studies on the thymus in Chagas' disease. I. Changes in the thymic microenvironment in mice acutely infected with *Trypanosoma cruzi*. Eur. J. Immunol. **19:** 1727–1733.

Schmitt D., Zambruno G., Staquet M.-J., Dezutter-Dambuyant C., Ohrt C., Brochier J., and Thivolet J. (1987). Antigenic thymus-epidermis relationships. Reactivity of a panel of anti-thymic cell monoclonal antibodies on human keratinocytes and Langerhans cells. Dermatologica **175:** 109–120.

Sun T.-T., Tseng S.C.G., Huang A.J.-W., Cooper D., Schermer A., Lynch M.H., Weiss R., and Eichner R. (1985). Monoclonal antibody studies of mammalian epithelial keratins. A review. Ann. N.Y. Acad. Sci. **455:** 307–329.

Takacs L., Savino W., Monostori E., Ando I., Bach J.-F., and Dardenne M. (1987). Cortical thymocyte differentiation in thymomas: Immunohistologic analysis of the pathologic microenvironment. J. Immunol. **138:** 687–698.

van Vliet E., Melis M., and van Ewijk W. (1984). Monoclonal antibodies to stromal cell types of the mouse thymus. Eur. J. Immunol. **14:** 524–529.

van de Wijngaert F.P., Rademakers L.H.P.M., Schuurman H.J., de Wegar R.A., and Kater L. (1984). Heterogeneity of epithelial cells in the human thymus. An ultrastructural study. Cell Tissue Res. **237:** 227–237.

